# The Past, Present, and Future in the Diagnosis of a Neglected Sexually Transmitted Infection: Trichomoniasis

**DOI:** 10.3390/pathogens13020126

**Published:** 2024-01-29

**Authors:** Alexandra Ibáñez-Escribano, Juan José Nogal-Ruiz

**Affiliations:** 1 Research Group Antiparasitic Epidemiology, Diagnostics and Therapy, PARADET, Complutense University of Madrid, 28040 Madrid, Spain; jjnogalr@ucm.es; 2 Department of Microbiology and Parasitology, Faculty of Pharmacy, Complutense University of Madrid, Plaza Ramón y Cajal s/n, 28040 Madrid, Spain

**Keywords:** trichomoniasis, STI, wet mount, culture, diagnosis, NAAT, point-of-care

## Abstract

More than one million curable sexually transmitted infections occur every day. *Trichomonas vaginalis* is one of the main infections responsible for these epidemiological data; however, the diagnosis of this protozoan is still mainly based on microscopic and culture identification. The commercialization of immunological tests and the development of molecular techniques have improved the sensitivity of classical methods. Nevertheless, the fact that trichomoniasis is a neglected parasitic infection hinders the development of novel techniques and their implementation in routine diagnosis. This review article shows the different methods developed to identify *T. vaginalis* in population and the difficulties in diagnosing male and asymptomatic patients. The importance of including this parasite in routine gynecological screening, especially in pregnant women, and the importance of considering *T. vaginalis* as an indicator of high-risk sexual behavior are also discussed.

## 1. Introduction

Sexually transmitted infections (STIs) remain a major public health concern. More than 30 pathogens can be transmitted through sexual intercourse, and nearly one million people are infected with a curable sexually transmitted pathogen every day [1,2]. The World Health Organization estimated an incidence of more than 377 million cases of chlamydia, gonorrhea, trichomoniasis, and syphilis in women and men during 2020 [3]. Although the incident cases of trichomoniasis are nearly 156 million [4], these epidemiological data may be underestimated due to the high number of asymptomatic patients [5], the low sensitivity of the preferred diagnostic methods used in many regions [6], and the fact that *Trichomonas vaginalis* infection is not a notifiable disease [7]. For all these, trichomoniasis has been included in the list of neglected parasitic infections (NPI) by the Center for Disease Control and Prevention (CDC) [8].

Trichomoniasis is characterized by a wide range of signs and symptoms associated with the inflammatory response triggered by the settlement of the parasite [9]. In women, about 75% of patients develop clinical manifestations. The most common are pruritus, local edema, erythema, dysuria, and/or a typical green, frothy, and malodorous vaginal discharge, among others [5,10,11]. In men, nearly 80% are asymptomatic; however, nongonococcal urethritis, epididymitis, or prostatitis may occur [5,10]. Although trichomoniasis has been considered as a “nuisance” infection [12,13], the complications and risks associated with this STI have led to its inclusion in the WHO Global Health Strategy on STIs for the period 2022–2030 [2]. *T. vaginalis* increases, by 1.5 times, the risk of acquiring HIV [14] but also favors its transmission due to the imbalance in the vaginal microbiome, the proinflammatory immune response, and the elevated vaginal pH [15]. In this scenario, coinfections with different urogenital pathogens are common among women with trichomoniasis, i.e., *Chlamydia trachomatis*, *Neisseria gonorrhoeae*, *Treponema pallidum*, human papillomavirus, or herpes simplex virus types 1 and 2 [16,17]. Other serious sequelae include pelvic inflammatory disease (PID) [2], adverse pregnancy outcomes [9,18], and infertility [9]. Moreover, several studies have associated trichomoniasis with an increased risk of cervical carcinogenesis [19,20,21]; notwithstanding, the association between *T. vaginalis* and prostate cancer remains under discussion [22,23,24]. 

This STI has been treated with metronidazole since 1959 [25]. Four decades later, the use of tinidazole was accepted [26,27,28], and recently the Food and Drug Administration has approved the use of secnidazole in the United States [29]. Likewise, in recent years, new recommendations on dosage in the treatment of infected women have been proposed to achieve a better rate of complete cure [28]. However, in spite of all this, almost 10% of clinical cases are resistant [30] and cross-resistance between 5-nitroimidazole drugs has been reported [31]. The absence of pharmacological alternatives to cope with treatment failure, hypersensitivity to 5-nitroimidazoles, or side effects [32,33] increases the risk of transmission and the development of chronic infection. Additionally, as trichomoniasis is highly asymptomatic and there are no diagnostic procedures to identify resistant and asymptomatic cases, the diagnosis based on syndromic management or microscopic identification of the parasite [34] hinders the correct management of the infected population. Therefore, the implementation of techniques with high sensitivity and specificity for use in routine and universal screening would reduce the incidence of this STI and, therefore, the risk associated with the acquisition of other pathologies, contributing significantly to this serious health problem.

In this review, we describe the different diagnostic techniques that have been used for the identification of *T. vaginalis* in female and male populations. In particular, we focus on the classical methods, based on microscopic identification such as wet mount and culture, as they have been the gold standard. Then, we revise the immunological and molecular techniques that have been recently developed. We discuss the information exposed with the aim of giving a general overview of what is happening in the diagnosis of this “neglected” STI, with special emphasis on the difficulty in low-income countries to implement the latest techniques. Finally, we highlight the growing trend of point-of-care (POC) techniques as an easy and fast strategy to improve the diagnosis of this NPI.

## 2. Diagnosis Based on Clinical Signs and Symptoms

Routine gynecological or urological examination does not include the identification of *Trichomonas*, so clinicians usually manage symptomatic patients who present different and nonspecific symptoms [10,11,35], such as vaginal discharge, odour and vulvar irritation. Physical examination of patients may show an erythematous vulva and vaginal discharge in 50% to 75% of cases [36]. A yellowish-green frothy discharge is a clinical sign suggestive of trichomoniasis [37,38]; however, this clinical manifestation is unusual. The most prevalent clinical presentation in patients includes vulvar pruritus and erythema, occasionally accompanied by oedema, dysuria, and dyspareunia [37,39]. Some studies highlight that vulvar irritation is more prominent in trichomoniasis than in bacterial vaginosis [36]. The pathognomonic sign of vaginal and/or exocervical punctate lesions can be observed by colposcopy in 45% of patients [36].

In addition to physical examination, determining the pH of vaginal secretions might facilitate the diagnosis. This pH increases above 4.7 in patients with trichomoniasis [40] or bacterial vaginosis. This biophysical parameter should be considered in the clinical context, considering that cervical fluids or semen may alter it.

The whiff test can also be performed. Briefly, the addition of a few drops of 10% KOH to the vaginal swab produces an unpleasant, fishy, amine-like odour. A positive odour test is observed in 75% of women with trichomoniasis and/or bacterial vaginosis [40], but not in those with vaginal candidiasis [36]. The high prevalence of asymptomatic patients and the absence of determinant clinical parameters imply the need for appropriate laboratory diagnostic methods to confirm *T. vaginalis* infection [5,36,41,42,43].

## 3. Classical Diagnostic Procedures: Microscopy

Diagnosis of trichomoniasis has traditionally consisted of taking a specimen from the patient and examining it immediately under the microscope [44,45] to identify the characteristic morphology and motility of the trophozoite. The most reliable specimens for the diagnosis of female trichomoniasis include endocervical and vaginal swabs and urine [46,47,48], while in male patients are urine, urethral swabs, and semen [48,49]. Cervicovaginal and urethral specimens are obtained with cotton swabs or polyester sponges [44,45,46].

Today, wet mount microscopy is the fastest and most widely used method for diagnosing trichomoniasis in resource-limited areas [50]. This method can have a specificity of 100%; nevertheless, it must be carried out quickly enough and at a temperature that does not impair the viability of the trophozoite. It is important to note that sensitivity values may decrease depending on the time elapsed from sample collection to microscopic examination [41]. Furthermore, this subjective method achieves a sensitivity that, depending on the experience of the technician, can range between 35–80% in comparison with the culture method [51,52,53]. In addition, a delay in specimen transport reduces the motility of trichomonads, which affects the procedure’s sensitivity [54,55]. This method is the most efficient diagnostic test, but its reliability and sensitivity are not optimal [52,53]. This low potential sensitivity contributes to the underdiagnosis of the disease.

Ideally, the saline wet mount preparations should be examined from the swab collected by the clinician, as well as inoculation of the samples immediately after collection in an appropriate culture medium [35,54]. Also, if samples are not immediately observed, they can be maintained in a suitable transport medium to avoid dehydration and redox potential changes. Stuart’s transport culture media and its modifications are the most recommended [56,57,58]. The average survival time in these transport culture media is approximately 24 h [59]. Inadequate transport or storage conditions may reduce the parasites viability and influence the wet mount technique sensitivity [41]. In this context, permanent staining techniques were developed as a complement to the direct examination of wet mount preparations [45].

### 3.1. Wet Smears

Microscopic observation of vaginal exudate diluted in saline is the routine procedure for the diagnosis of female trichomoniasis; however, epithelial cells and polymorphonuclear leukocytes in the samples may interfere with the parasite’s flagellar motility. The sensitivity of this technique is highly variable due to multiple causes, such as the type of sample, the number of viable organisms, and the delay between the obtention and the microscopic diagnosis, among others [51,52,53,60,61]. 

Regarding male samples, Feinberg and Whittington observed a greater sensitivity when direct microscopy was used with urethral material than culture methods [60]; however, there are discrepancies in other reports [62].

### 3.2. Staining Techniques

Different stains have been developed to increase the sensitivity of direct examination. Stained smears can be preserved without loss of diagnostic reliability due to adequate fixation and be observed later [45]. The most frequently used stains include Papanicolaou, Giemsa, and acridine orange [63,64,65,66,67,68,69]. Furthermore, less-well-known stains have also been tested, including Leishman, periodic acid–Schiff, and Fontana–Masson stains [67,68,69]. The Giemsa stain is perhaps the most accessible in the laboratory and has been used for more than 100 years in trichomoniasis diagnosis [70]. In these preparations, the nucleus of trichomonads stains purplish red and the cytoplasm is light red, pink, or bright blue, depending on pH, with a darker staining nucleus that may be oval or spindle-shaped. Sometimes, axostyle and flagella can be observed [71]. Generally, microscopic examination of Giemsa-stained smears is more effective in detecting infections than wet smear microscopy [72,73,74] and may have a sensitivity near that of culture [67,72,75].

Other stains such as safranin, methylene blue, and malachite green, which do not stain trophozoites, can act as counterstains [76,77,78]. Fluorescein can also be used to observe wet mount slides under an ultraviolet light microscope [79]. Thus, acridine orange for fluorescence-based detection of *T. vaginalis* has also been suggested by other authors as it exhibits a greater sensitivity than Giemsa staining but requires UV fluorescent light microscopy [47,80]. However, these staining methods have not been convincingly demonstrated to improve the detection rate of trichomonads in secretions and are not recommended for routine clinical diagnosis. 

In Papanicolaou smears of cervicovaginal material, *T. vaginalis* exhibits an ovoid structure and an approximate size of 10–30 μm with a greenish grey cytoplasm which contains very small eosinophilic granules, and the eccentric nucleus stains blue. The sensitivity and specificity of this stain vary depending on the experience of the microscopist [41,77,81] as shown in Table 1.

The traditional diagnostic methodology is easy to perform; however, these techniques have the disadvantage of not being very sensitive and require careful observation by expert microscopists.

### 3.3. Culture

Liquid or broth culture of a clinical specimen (cervicovaginal, urethral, or urinary sediment) for microscopic observation has been considered the gold standard technique for the diagnosis of trichomoniasis, due to its sensitivity, simplicity, and the relatively low inoculum requirement (300 trichomonads/mL) [82]. Several media have been described for the *T. vaginalis* culture: Kupferberg, Kupferberg STS, Hirsch, Trichosel, Modified Diamond, Lash serum, or the most recent, called InPouch^®^ TV [83,84,85]. However, the most common are Diamond (TYM), modified Diamond, or Roiron^®^ [41]. 

Diamond’s medium requires refrigeration at 4 °C for storage but should be at room temperature before specimen inoculation. Samples should be inoculated immediately into the culture medium, at least 1 h after collection, and incubated at 37 °C in anaerobic conditions (5% CO_2_). This should be followed by daily examination for 3–7 days until viable trichomonads are observed [41,82]. Longer incubation times are often required in male specimens to allow the growth of a detectable number of organisms [6].

Thus, this methodology is simple and inexpensive, but requires the direct microscopic examination during a long period [49] in which infected patients may continue to transmit the infection [86]. Moreover, there are inherent limitations to culture diagnosis, e.g., culture contamination with vaginal microbiota (bacteria or yeasts) can be very frequent [87,88]. Nevertheless, sensitivity rates can rise to nearly 95% depending on the sample and the medium used (Table 1). 

To enhance the acceptance of culture diagnosis, a good procedure is the so-called delayed inoculation, a method that combines both techniques: first, the fresh sample for direct examination and, if negative, its incubation in culture medium for 2–5 days [89]. Regarding this, InPouch^®^ TV is a self-contained system which permits both immediate examination and culture in a single device of vaginal, urethral, and urine samples. The sensitivity is comparable to that obtained with wet smears and culture specimens [90,91]. The transparent oxygen-resistant plastic can be examined directly under the microscope, allowing daily examination of the specimen without removing it from its culture medium. InPouch^®^ TV can be kept at room temperature, and even inoculated pouches can remain at room temperature for up to 48 h before incubation at 37 °C [41]. Levi and coworkers demonstrated that the InPouch^®^ TV system was as sensitive as modified Diamond’s medium for *T. vaginalis* detection [92]. Borchardt and collaborators demonstrated that this system is more sensitive than modified Diamond’s medium or Trichosel medium [93]. However, InPouch^®^ TV continues to be a procedure that requires observation of the sample for several days, not a rapid diagnostic technique [41,49]. 

**Table 1:** Relevant characteristics of the techniques used in the direct diagnosis of *T. vaginalis*.

Type of Diagnosis	Test	Sensitivity (Se) Specificity (Sp)	Advantages	Disadvantages	Ref.
Microscopy	Wet smears	Se: 35–85% Sp: 100%	Fast, simple, and inexpensive.	Sensitivity depends on the skills of the microscopist. Not applicable to male specimens.	[51,52,53,60,61]
Staining	Giemsa	Se: 80% Sp: 99.4%	Fast, simple, and inexpensive. Improved sensitivity vs. wet smears Stain used in Pap smears. Fast, simple, and inexpensive.	Staining specialists required to improve sensitivity.	[67,72,75]
	Acridine orange	Se: 100% Sp: 100%			[45]
	Papanicolaou	Se: 60–95% Sp: 98–100%			[41]
Culture	Diamond medium	Se: 56–95.8% Sp: 100%	Improved sensitivity vs. wet smears. Less handling, simple, easy to transport	Requires equipment and laboratory specialist. Risk of pathogen contamination and false negatives. Long incubation period.	[41,90,91]
	InPouch^®^	Se: 92% Sp: 98%			[92,93]

Therefore, it was necessary to develop and implement more rapid, sensitive, and specific tests that allow early diagnosis in at-risk populations and even the identification of asymptomatic cases. These new methods, some of them POC techniques, are currently being implemented in many diagnostic services [91].

## 4. Immunodiagnosis

### 4.1. Latex Agglutination Techniques

One of the techniques used for the serodiagnosis of trichomoniasis is latex agglutination. Among the different commercial tests available for the detection of *T. vaginalis*, the Kalon TV latex^®^ agglutination test stands out. The kit includes a suspension containing latex particles sensitized with rabbit anti-*T. vaginalis* [94], which must be brought into contact with a drop of diluted sample of vaginal exudate from the patient [95]. In addition, some laboratories have developed agglutination kits that identify simultaneously other microorganisms that cause vaginal infections, such as *Candida* spp. [96,97]. These tests use two different reagents, one sensitized with anti-*T. vaginalis* antibodies, and another with anti-*Candida* spp. [97]. Latex agglutination methods offer a better sensitivity than fresh examination and are comparable to culture [98], reaching up to 100% in more recent studies, becoming a technique with a quite acceptable cost-effectiveness ratio (Table 2) [95]. Therefore, agglutination tests present quite a few advantages; its specificity is comparable to that of cultivation, it takes less than three minutes to offer results, is very simple to carry out, and does not require a microscope or any special equipment, nor specialized personnel [98]. However, in those symptomatic patients with a negative agglutination result, a different diagnostic method must be performed, with the aim of (i) avoiding overlooking possible infections after a false negative result and (ii) confirming true positives [95,98]. This is due to their low positive predictive value, which is only 60% [95]. It is worth mentioning that these tests cannot be used for diagnosis in men, as they can only be performed on vaginal exudate samples [95,98].

### 4.2. ELISA

The indirect ELISA (enzyme-linked immunosorbent technique assay) is a serodiagnostic test that detects specific antibodies revealing the exposure of the patient to the parasite. This procedure has been reviewed in numerous scientific works and is one of the most established serological techniques due to its sensitivity and specificity [99,100]. To avoid cross-reactions and false positives, the epitopes recognized by the patient’s antibodies must be specific to *T. vaginalis* and not present homology with those of other microorganisms, nor with the host’s own cells [101,102]. In relation to the antigens of *T. vaginalis*, the following stand out for their immunogenicity: α-actinin, α-enolase, aldolase, and glyceraldehyde-3-phosphate dehydrogenase [102].

With the aim of designing an ELISA test with the best sensitivity and specificity, some researchers have explored the possibility of obtaining synthetic recombinant peptides, which contain more than one epitope that can be recognized by a greater number of specific antibodies [102]. Over time, these recombinant chimeric proteins have been improved, eliminating those that share certain homology with proteins from other organisms, thus increasing the sensitivity of this technique for screening patient sera [102,103,104]. The synthesis of recombinant chimeric proteins can be performed using bacterial plasmids, being much more advantageous and efficient than using whole cells or cell lysates to avoid cross-reactions of common epitopes [102]. However, when recombinant antigen technology is not available, it is common to use a lysate of different strains of the parasite to perform the indirect ELISA and quantify the seropositivity of patients. Serum is the most suitable sample for the detection of anti-*T. vaginalis* antibodies, since IgG are found in a higher percentage in serum than in vaginal exudate, while IgM are not even detected in some studies [105]. Moreover, the use of serum makes this methodology suitable for the diagnosis of trichomoniasis in both sexes [103]. However, if the aim is to detect IgA antibodies, the predominant isotype in seromucous secretions, vaginal and endocervical exudate are more appropriate samples [106].

This methodology is only capable of detecting relative recent past infection, as antibody levels can become undetectable around 6–12 months after infection [107], being inadequate to discriminate between an active infection (acute or chronic) and a past exposure. Nevertheless, it is a sophisticated technique that requires specialized personnel, as well as laboratory equipment that may not be available in some areas. 

On the other hand, the sandwich ELISA technique uses specific capture antibodies immobilized on the microtiter plate for the detection of *T. vaginalis* antigens [99,108]. Recent studies have confirmed that this type of ELISA has a sensitivity of 88.9% and a specificity of 97.1% in the detection of *T. vaginalis* antigen when compared with culture as the reference method [109]. These values have already been defined in previous studies, using monoclonal antibodies for the detection of parasitic surface antigens present in vaginal exudate samples [110]. However, beyond these excellent data, this method has a false positive rate of 2.9%, as shown in Table 2. This could be due to the detection of nonviable trophozoites, something that occurs less frequently in culture, which is based on the identification of organisms viable with motility [109].

### 4.3. Immunofluorescence

Immunofluorescence techniques are not routinely used for diagnosis. In recent years, direct immunofluorescence (DIF) has been used for research purposes, using fluorophore-labeled monoclonal antibodies as a conjugated to detect *T. vaginalis* antigens [111]. DIF is commonly used for the study of morphological and metabolic characteristics of the parasite [111,112], showing higher sensitivity and specificity than direct techniques [113]. 

**Table 2:** Relevant characteristics of the techniques most commonly used in the immunodiagnostic of *T. vaginalis*.

Test	Trade Name (Manufacturer)	Sensitivity (Se) Specificity (Sp)	Advantages	Disadvantages	Ref.
Agglutination	Kalon^®^ TV latex agglutination test (Kalon Biological) Darari and others	Se: 98.9% Sp: 92.1%	Fast, simple, and inexpensive.	In case of negative results, other methods must be carried out. Not designed for male samples.	[98,99]
		Se: 70% Sp: 96%			
Indirect ELISA		Se: 71–73% Sp: 96.3%	Detects past infections. Admits male specimens.	Requires complex and expensive equipment. Low sensitivity.	[100]
Direct ELISA		Se: 88.9% Sp: 97.1%	Good sensitivity and specificity.	Detects only active infections. High rate of false positive rate.	[109]
DIF *		Se: 96% Sp: ND	Good sensitivity.	Requires complex and expensive equipment.	[114]
IC *	OSOM^®^ TV test (Sekisui Diagnostics)	Se: 83–90% Sp: 98.9%	Fast, and simple. Good sensitivity and specificity.	Not suitable for asymptomatic patients.	[34]

* Abbreviations: DIF: direct immunofluorescence; IC: immunochromatography.

The use of immunofluorescence techniques for the diagnosis of *T. vaginalis* can offer certain advantages, being a very specific and accurate test, providing easy interpretation of the results. However, it is quite sophisticated and requires a fluorescence microscope, as well as trained personnel for its management (Table 2) [80,114]. Some authors consider that it could be a useful technique for confirmation of negative results after direct microscopic examination of the sample or culture, for example [113].

### 4.4. Immunochromatography

As mentioned above, POC tests are being implemented to improve syndromic management and classical techniques to facilitate prompt diagnosis and treatment. These tests offer results in minutes and are easier to perform [115]. They do not need sophisticated equipment and their simple procedures can be performed with minimal training [116]. The most used POC tests are immunochromatography type, rapid molecular assays, or based on agglutination reactions [115]. This is the case of the OSOM^®^ *Trichomonas* test, a lateral flow immunoassay capable of detecting an adhesin of *T. vaginalis* by using monoclonal antibodies immobilized on the nitrocellulose membrane [117]. This FDA-cleared test also uses another antibody conjugated with blue-colored particles, so when the immune complex is formed, a line of this color shows a positive result. As in many immunochromatographic tests, the control line, which should always appear, is colored red in this test [118]. OSOM^®^ *Trichomonas* test exhibits a sensitivity between 83–90%, comparable to NAAT (nucleic acid amplification test), and a specificity in vaginal exudate samples > 98.8% (Table 2). Furthermore, this test can be also performed by the patient at home [34].

Alderete and Chan (2023) have recently developed a POC using the MedMira Rapid Vertical Flow (RVF^®^) technology. The antigen used is a 72.4 kDa truncated version of α-actinin called ACT::SOE3 that can be detected by specific antibodies present in human sera. This immunochromatographic device stands out because it can be used in both men and women with trichomoniasis [119].

## 5. Molecular Diagnosis

Different commercial and “in-house” PCR-based assays to detect *T. vaginalis* nucleic acids have been implemented in recent years. PCR techniques provide a remarkably higher sensitivity than microscopic methods; however, trained staff, equipment, and cost of reagents are the main drawbacks associated with most of these techniques [91]. Prior to the development of the nucleic acid amplification test (NAAT), wet mount and culture methods were the gold standard for trichomoniasis diagnosis; however, in the last decades, molecular techniques have become the most appropriate techniques for the diagnosis of this infection when possible [120].

There are a dozen FDA-approved assays for the detection of *T. vaginalis* by NAAT. Some of them also comply with European Standards, as reflected in Table 3 [120,121]. These tests can detect DNA or RNA of the parasite in symptomatic and asymptomatic patients, with a high range of sensitivity and specificity, depending on the assay.

All the mentioned tests identify specimens in clinician-collected vaginal swabs; however, the differences between the following techniques are mainly based on the method used for the identification of nucleic acids, the possibility of detecting more than one urogenital pathogen, and the type of sample that can be analyzed (Table 4). For example, the Xpert^®^ TV assay (Cepheid, Sunnyvale, CA, USA) stands out because it can be used in self-collected vaginal swabs, as well as female and male urine [122]. This system includes a kit designed to collect, preserve, and transport *C. trachomatis*, *N. gonorrhoeae*, and *T. vaginalis* specimens that can be analyzed posteriorly in two different assays using the same real-time PCR (RT-PCR) platform: Xpert^®^ CT/NG and TV Assays. The studies of microbial interference using a panel of 124 microorganisms revealed no cross-reaction apart from *Trichomonas tenax* when concentration was higher than 200 cell/mL [121]. The Aptima^®^ *T. vaginalis* Assay (Hologic, Bedford, MA, USA) identifies a specific region of the small ribosomal subunit in female endocervical and vaginal samples, which are collected in tubes with transport media (PreservCyt^®^) that release and protect RNA during storage. Then, the rRNA is capture by specific oligomers associated with magnetic particles, and, posteriorly, the amplification is performed by transcription-mediated amplification (TMA). The Aptima^®^ system is already used to discriminate between *T. vaginalis*, *C. glabrata,* and *Candida* spp. (i.e., *C. albicans*, *C. dubliniensis*, *C. parapsilosis*, *C. tropicalis*) as well as *Chlamydia trachomatis* and *Neisseria gonorrhoeae* using the same Panther^®^ platform [121]. All the devices developed by Hologic are based on the identification of specific rRNA targets of the different pathogens mentioned. Curiously, BD Affirm^TM^ VPIII (Becton Dickinson &Co., Franklin Lakes, NJ, USA) also detects rRNA from three urogenital pathogens: the protozoan *T. vaginalis*, the bacteria *Gardnerella vaginalis,* and the yeast *Candida* spp. (including *C. albicans*, *C. glabrata*, *C. kefyr*, *C. krusei*, *C. parapsilosis*, and *C. tropicalis*). Female samples should be collected and transported at room temperature or refrigerated (2–8 °C) using a specific collection swab and a transport system provided by the company that conserves the samples up to 72 h [134]. The comparison studies between the Xpert^®^ TV assay and Aptima^®^ system with endocervical swabs, patient-collected vaginal swabs, and urine samples have demonstrated similar sensitivity and specificity values, slightly higher than those detected with the InPouch system [122], while the comparison performed by Andrea and Chapin (2011) of the two rRNA assays (Aptima^®^ and Affirm VPIII) for the detection of *T. vaginalis* in 41 positive and 740 negative samples indicated that the Aptima^®^ system was significantly more sensitive (41/41) than the Affirm^TM^ VPIII device (26/41) [126]. Roche has also developed an NAAT device to identify *T. vaginalis* and *M. genitalium* by targeting multicopy regions of rRNA from the parasite by RT-PCR. The Cobas^®^ TV/MG highlights the large number of samples that can be used for the diagnosis of these two urogenital pathogens, including self-collected vaginal swabs, endocervical samples, liquid-based cytology specimens in PreservCyt solution, and urine from both male and female patients. As occurs with other NAAT systems, for correct transport and DNA processing, the swab and urine samples must be taken using a sample kit provided by the laboratory [130]. Two other cleared tests that can detect *T. vaginalis* in the same type of samples as indicated for Cobas^®^ are BD CTGCTV2 (Becton Dickinson & Co.) and Alinity m STI Assay (Abbott). Both systems simultaneously detect *C. trachomatis*, *N. gonorrhoeae,* and *T. vaginalis* [121,135]. Moreover, both include an automated DNA extraction prior to the quantitative real-time PCR all in the same benchtop instrument [127]. The only difference is that Alinity also identifies rRNA from *Mycoplasma genitalium* [119]. BD Diagnostics has developed another NAAT system for the detection of *T. vaginalis* in self- and clinician-collected vaginal swabs, endocervical swabs, and female urine samples. The BD ProbeTec Q^x^ Assay needs a prewarming step prior to processing the sample onto the Viper System which automatically performs the extraction and amplification steps based on the strand displacement amplification (SDA) technique. The DNA target used for the diagnosis of *T. vaginalis* on female patients is the gene that encodes the parasitic adhesion protein AP65 [120,124]. This platform can be used for the detection of other STI pathogens such as *N. gonorrhoeae* or *C. trachomatis* [124].

Although Solana^®^ *Trichomonas* assay identifies only *T. vaginalis* in samples, it should be highlighted that it has been developed as a POC test for qualitative detection of *T. vaginalis* using isothermal helicase-dependent amplification (HDA) for the detection of a multicopy DNA fragment of the parasite in the sample [131]. In this sense, another POC using HDA technology has been developed by Quidel Corporation: the AmpliVue^TM^ *Trichomonas* Assay. The main differences between both systems are the samples that can be used and the automatization of the detection technique. For the former, clinician-collected vaginal swabs and female urine samples can be employed. The method consists of two steps: first, the sample is included in a lysis tube and heat-treated at 95 °C. Then, an aliquot is added to the reaction tube that contains the HDA reagents for the automated detection of the *T. vaginalis* DNA target in the Solana instrument [131]. No cross-reaction with 47 different microorganisms, including bacteria, virus, and protozoa, or interference has been detected [121], while for the latter, only clinician-collected vaginal swabs can be used. The management of the sample by heat treatment and the isothermal DNA amplification is performed as mentioned above; however, the identification of the parasitic DNA target is effectuated in a cassette after the HDA reaction. In other words, the AmpliVue^TM^ test needs a third step that includes a vertical-flow strip for the colorimetric detection of the parasitic DNA. Both POC methods have presented similar sensitivity and specificity as other NAAT assays such as Aptima^®^ [34,132]. The last POC system, based on NAAT, is the Visby Medical Sexual Health Test, a compact device that can detect *C. trachomatis*, *N. gonorrhoeae,* and *T. vaginalis* ins self-collected female vaginal swabs using a specific collection kit in 30–50 min [121,133].

The development of detection systems that include (i) the automatization of DNA extraction, (ii) the identification of more than one pathogen in the same sample, or (iii) the ability to process self-collected samples, endocervical swabs, or/and male urine samples can significantly improve the sensitivity and specificity of classical diagnostic methods becoming much more useful techniques for the diagnosis of STIs [2]. However, the increased cost of these techniques for the diagnosis of *T. vaginalis*, which continues receiving less attention [13], could explain why their implementation is reduced. Fortunately, the development of diagnostic assays that can be implemented in the systems used for the detection of other STIs have become an interesting tool for laboratories like Abbott, Roche, Hologic/Gen-Probe, BD, or Cepheid to invest in the development of NAAT tests to identify *T. vaginalis* (Table 3 and Table 4).

**Table 4:** Types of samples and other sexually transmitted pathogens that can be detected in the same NAAT platform. PC-VS: patient-collected vaginal swab; CC-VS: clinician-collected vaginal swab; ES: endocervical swab; US: urine sample; PC-E: endocervical specimens collected in PrservCyt^®^ solution; MS: meatal swab.

Assay	Test	Samples	STI	Ref.
Xpert^®^ *Trichomonas vaginalis*	RT PCR	Female SC-VS, CC-VS, ES and US Male US	*C. trachomatis*, *N. gonorrhoeae*	[122,131]
APTIMA^®^ *Trichomonas vaginalis*	TMA	Female SC-VS, CC-VS, ES, PC-E and US	*C. trachomatis*, *N. gonorrhoeae*, *Candida* spp.	[123]
BD Affirm^TM^ VPIII	rRNA hybridization	Female CC-VS, US	*G. vaginalis*, *C. albicans*	[126]
BD CTGCTV2	RT PCR	Female SC-VS, CC-VS, ES, PC-E and US Male US	*C. trachomatis*, *N. gonorrhoeae*	[127]
BD ProbeTec Q^x^ Assay	SDA	Female SC-VS, CC-VS, ES and US	*C. trachomatis*, *N. gonorrhoeae*	[136]
Alinity m STI	RT PCR	Female SC-VS, CC-VS, ES and US Male US	*C. trachomatis*, *N. gonorrhoeae*, *M. genitalium*	[121,129]
Cobas^®^ TV/MG	RT PCR	Female SC-VS, CC-VS, ES, PC-E and US Male US and MS	*M. genitalium*	[130,137]
Solana^®^ *Trichomonas* Assay	HDA	Female CC-VS and US		[131]
AmpliVue^TM^ *Trichomonas* Assay	HDA	Female CC-VS		[131]
Visby Medical Sexual Health Test	RT-PCR	Female SC-VS	*C. trachomatis*, *N. gonorrhoeae*	[133]

In addition to the cleared systems mentioned above, different RT-PCR assays for the simultaneous detection of more than one STI pathogen (including *T. vaginalis*) have also been published. Recently, different research groups have designed an in-house multiplex RT-PCR for the diagnosis of up to nine pathogens (*C. trachomatis*, *N. gonorrhoeae*, *T. vaginalis*, *C. albicans*, *Mycoplasma hominis*, *M. genitalium*, *Ureaplasma urealyticum*, *U. parvum*, *Gardnerella vaginalis,* and human herpes viruses) with sensitivity and specificity values > 91% and an estimated cost per test of nearly USD 3 [138,139]. Also, different groups have developed loop-mediated isothermal amplification (LAMP) assays for the detection of *T. vaginalis*. This methodology presents a high sensitivity and specificity, it can be used with urine and genital samples, and can amplify DNA with only a heat block or water bath in less than 130 min. However, it is not free from contamination and can give false positives [140,141].

Moreover, other research groups are focusing on the development of novel systems based on the use of aptamers for the diagnosis of *T. vaginalis* in vaginal samples. This novel method targets the detection of the parasitic immunogenic protein AP65 and could become an interesting tool based on its potential use in microtiter plate and lateral flow assays [142].

## 6. Discussion

Sexually transmitted infection is a global public health challenge with an increasing trend in the last years (58.15% since 1990) [143]. Of the different pathogens that can be transmitted by sexual intercourse, *T. vaginalis* causes the most prevalent nonviral sexually transmitted infection worldwide, mainly affecting the most disadvantaged populations in both developed and low-income countries [4,144,145]. Its prevalence is underestimated because it is not a notifiable disease. This is compounded by the lack of routine protocols to identify asymptomatic patients, the limited sensitivity of some diagnostic methods, and the absence of pharmacovigilance systems to detect resistance phenomena. In this context, many researchers, and organisms such as the CDC have classified trichomoniasis as a “neglected” disease [8,144,145,146].

In summary, the recommended procedures for the diagnosis of trichomoniasis, according to Eastern European [147] and CDC [148] guidelines, are as follows: (i) diagnostic screening in all patients with any vaginal discharge, in high-prevalence environments (STI clinics), and for the asymptomatic population with high risk of infection (e.g., persons with multiple sexual partners, prostitution, drug abuse, or history of STIs) and (ii) employ NAAT if direct microscopic examination fails. According to the updated CDC guidelines for STI diagnosis, culture would no longer be considered the gold standard for diagnosis of *T. vaginalis* infection if alternative molecular methods are available [149].

The priority population groups for preventing STI infections have been changing in the last decades [120], due to the evolution observed in social/sexual behavior. This may explain why the age of first sexual intercourse has been reduced, as well as the perception of certain sexual behaviors as nonrisky (i.e., the use of condoms), increasing exposure to STIs [146,150,151]. Thus, sexual education and the inclusion of the main STI pathogens in the gynecological revisions could be interesting tools for the reduction of these infections.

This review notes the recent development of novel diagnostic techniques with higher sensitivity and specificity than those based on microscopic identification (i.e., wet mount, Papanicolaou, or Giemsa stain and culture). However, the highest prevalence rates of trichomoniasis are associated with low- and middle-income countries and regions [4], where the diagnostic protocols are frequently chosen based on their low cost and ease of use, in which no specialized equipment or personnel are required. Although wet mount diagnosis is easy and inexpensive, its main limitations are (i) the microscopic visualization of the samples must be taken within 20 min of its collection, (ii) the reduced sensitivity, and (iii) the fact that it is only possible for vaginal swab samples and urine [93,123].

The association between trichomoniasis and the risk of acquiring other pathogens through sexual intercourse has been demonstrated [14,16,17,152]. In this context, *T. vaginalis* could be used as a marker of high-risk sexual behavior. Considering that more than 746 new cases of HIV could be imputable to *T. vaginalis* [153], the diagnosis and treatment of patients with trichomoniasis becomes an interesting public health strategy for the control of STIs. This proposal becomes even more important if we take into consideration that in relation to the economic burden, the total costs of nonviral STIs in the United States are being estimated as USD 0.4 billion, with an average lifetime medical cost per *T. vaginalis* infection of USD 5 for men and USD 36 for women [149,154].

Therefore, it should be a priority to implement methods that could simultaneously diagnose multiple STI pathogens with technologies that could be applied in low-income regions. In this sense, the POC tests seem to be the best way to cope with this necessity. Currently, four POC devices have been developed for *T. vaginalis* diagnosis: one based on the detection of a parasitic antigen protein (OSOM^®^ *Trichomonas* test) and the other four in DNA detection (Solana^®^, AmpliVue^TM^, GeneXpert and Visby Medical Sexual Health Test). The first one is a low-complexity dipstick test that can be performed in less than 15 min without specific equipment, while Solana^®^ and AmpliVue^TM^ assays require small heat blocks for the lytic and the isothermal amplification steps [121,131,132].

In addition, another disadvantage that must be overcome in many of the diagnostic techniques described is that many of them are not suitable for male samples. As men are mainly asymptomatic carriers, the recommendations, rutinary test, and methods developed for these patients are scarce [137,155]. Wet mount of urine or urethral samples is inexpensive and easy to perform; however, the main limitation is its reduced sensitivity due to the limited time between sample collection and the observation using the microscope. The identification must be performed within 20 min before collection as not-motile *T. vaginalis* can be difficult to recognize by microscopy [123,156]. Fortunately, in recent years, some NAAT devices have been able to identify *T. vaginalis* in both male urine samples and meatal swabs, i.e., GeneXpert assay, Cobas^®^ TV/MG, BD CTGCTV2, or Alinity m STI Assay. It is important to highlight that all these devices can identify other STIs, as shown in Table 4. Thus, these detection systems, together with POC, are becoming the most powerful and useful tools to correctly identify this parasitosis in the population.

## 7. Conclusions

In conclusion, this article reviews the different diagnostic approaches to trichomoniasis in different circumstances. While in developed countries, nucleic acid amplification testing (NAAT) should displace direct examination of wet smears and culture, in middle- and low-income regions, this is not possible due to the equipment required in some molecular techniques. In addition, skilled laboratory technicians are also required in many cases. These requirements constitute a serious challenge in low-income countries, due to the high economic cost of providing adequate facilities and training specialists. In these regions, the diagnosis of trichomoniasis relies on anamnesis, clinical diagnosis supported by physiological test (i.e., vaginal pH or whiff test), and direct examination. Considering the importance of this STI as a marker of high-risk sexual activity, the implementation of affordable and easy-to-use POC techniques (i.e., OSOM^®^ *Trichomonas* test, GeneXpert, Visby Medical Sexual Health Test, or LAMP techniques, among others) opens the door for the future management of this “neglected” STI.

## Figures and Tables

**Table 3:** Sensitivity and specificity ranges of the nucleic acid amplification test (NAAT) approved by the U.S. Food and Drug Administration. Sens: sensitivity; Spec: specificity: CE-IVD: devices with the Conformité Européene marking for in vitro diagnostic medical devices.

Assay	Manufacturer	Average Sens. % (Range) *	Average Spec. % (Range) *	Approval Status	Ref.
Xpert^®^ *Trichomonas vaginalis*	Cepheid	Female: 99.5–100 Male: 97.2	Female: 99.4–99.9 Male: 99.3	FDA CE-IVD	[120,122]
APTIMA^®^ *Trichomonas vaginalis*	Hologic/Gen-Probe	98.1 (96.6–100)	98.3 (96.4–100)	FDA CE-IVD	[123,124,125]
BD Affirm^TM^ VPIII	Becton Dickinson	63.4 (55.0–65.4)	99.9 (99.4–100)	FDA	[123,126]
BD CTGCTV2	Becton Dickinson	Female (86.6–100) Male (97.9)	>98.7	FDA	[121,127]
BD ProbeTec Q^x^ Assay	Becton Dickinson	98.3 (93.93–99.53)	99.0 (98.01–99.53)	FDA CE-IVD	[120,124]
Alinity m STI	Abbott	Female: 95.6–99.7 Male: 98.7	Female: 96.9–99.4 Male: 99.2	FDA	[128,129]
Cobas^®^ TV/MG	Roche	Female: 100	Female: ≥99.2	FDA CE-IVD	[130]
Solana^®^ *Trichomonas* Assay	Quidel	98.0–100	97.9–98.9	FDA	[131]
AmpliVue^TM^ *Trichomonas* Assay	Quidel	96.9–100	97.0–98.9	FDA	[131,132]
Visby Medical Sexual Health Test	Visby Medical	95.5–99.9	95.8–97.7	FDA	[122,133]

* In case of many samples, the % indicates the lowest and highest value observed.
